# Supplementary material for: Molecular Landscape of Anti-Drug Antibodies Reveals the Mechanism of the Immune Response Following Treatment With TNFα Antagonists
Source: Front Immunol. 2019 Dec 18;10:2921. doi: 10.3389/fimmu.2019.02921 (PMC6930160; doi:10.3389/fimmu.2019.02921)
Supplement: Supplementary file 1 [file Data_Sheet_1.pdf]

Supplementary material

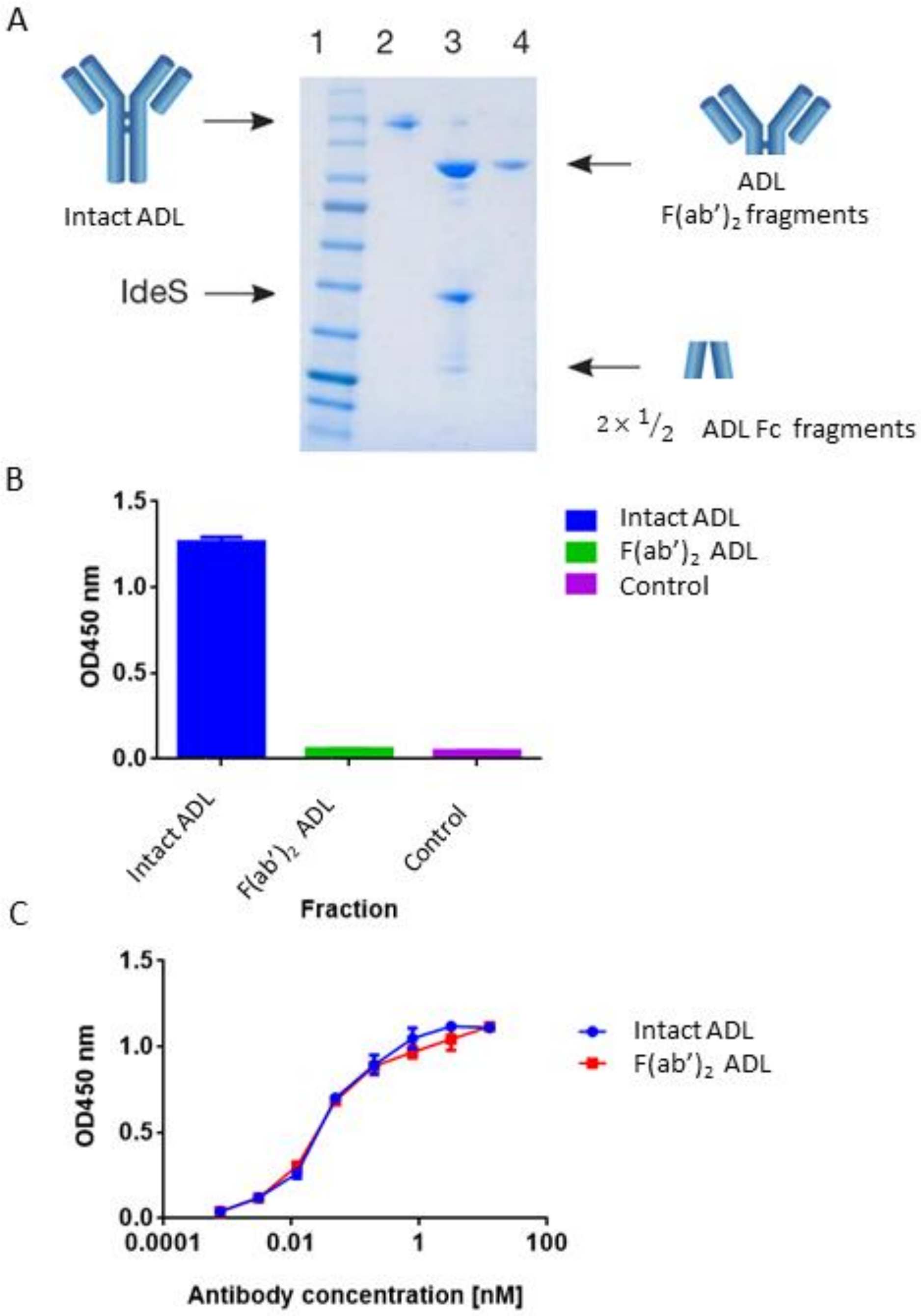

Figure S1: **ADL digestion and ADL-F(ab')$_2$ purification**. (A) SDS-PAGE analysis of intact ADL (lane 2), following IdeS digestion (lane 3) and purified ADL-F(ab')$_2$ following a 2-step affinity chromatography purification including protein A and kappaSelect columns (lane 4). (B) Presence of ADL-Fc and intact ADL traces was measured by direct ELISA were intact ADL and purified ADL-F(ab')$_2$ were compared to a control antigen (streptavidin) as coating agents followed by direct incubation with an anti-Fc HRP conjugate at the detection phase. (C) The functionality of the recovered ADL-F(ab')$_2$ was confirmed by ELISA and compared to intact ADL. The ELISA setup included TNFα as the coating agent and anti-κ HRP conjugate at the detection phase. For panel B–C, triplicate averages were calculated as mean, with error bars indicating s.d.

13

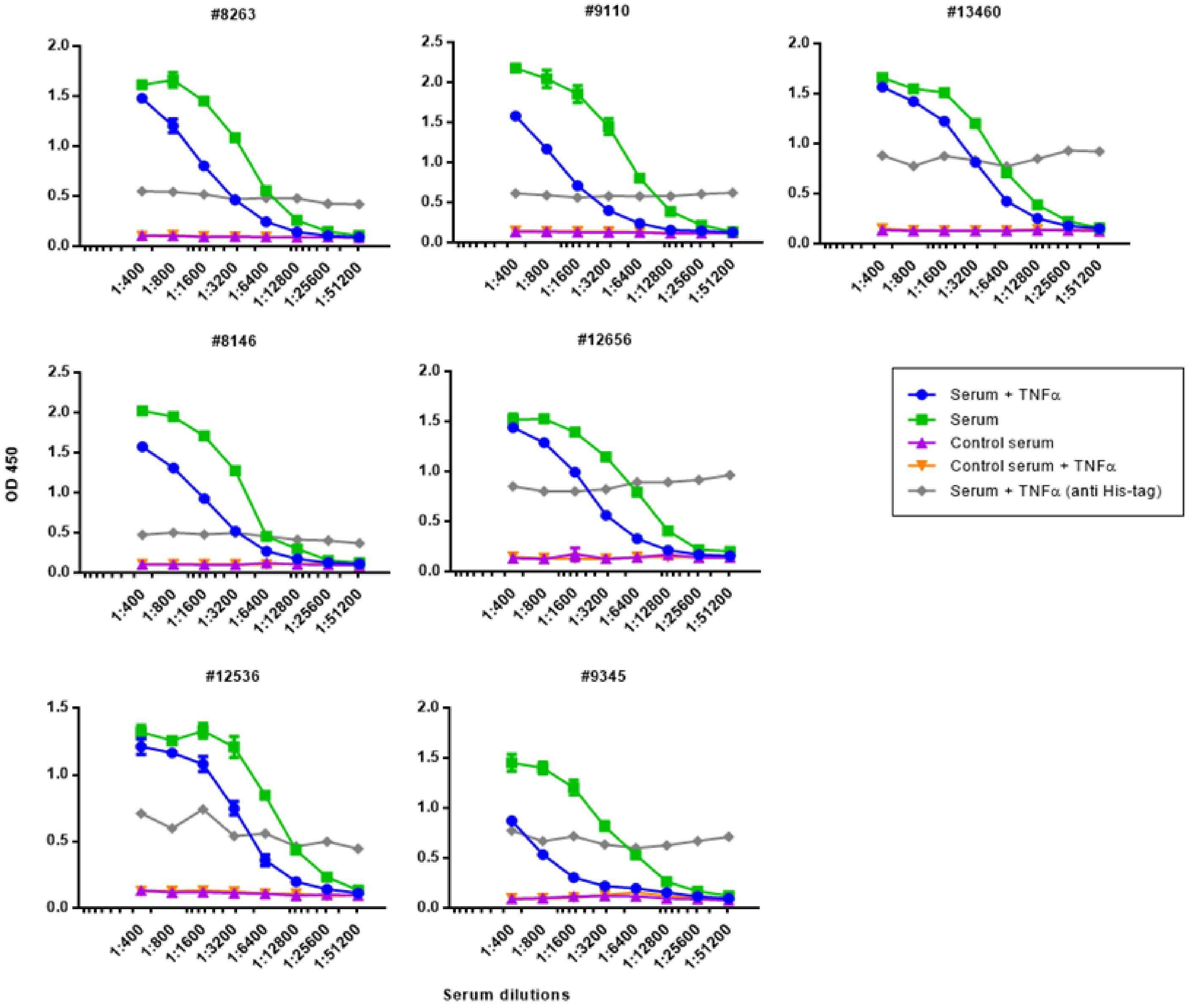

Figure S2: Determination of ADA neutralization index in patients treated with ADL. The reduction of the signal obtained when *nt*ADA are present in serum was indicative for sera with high neutralizing capacity.

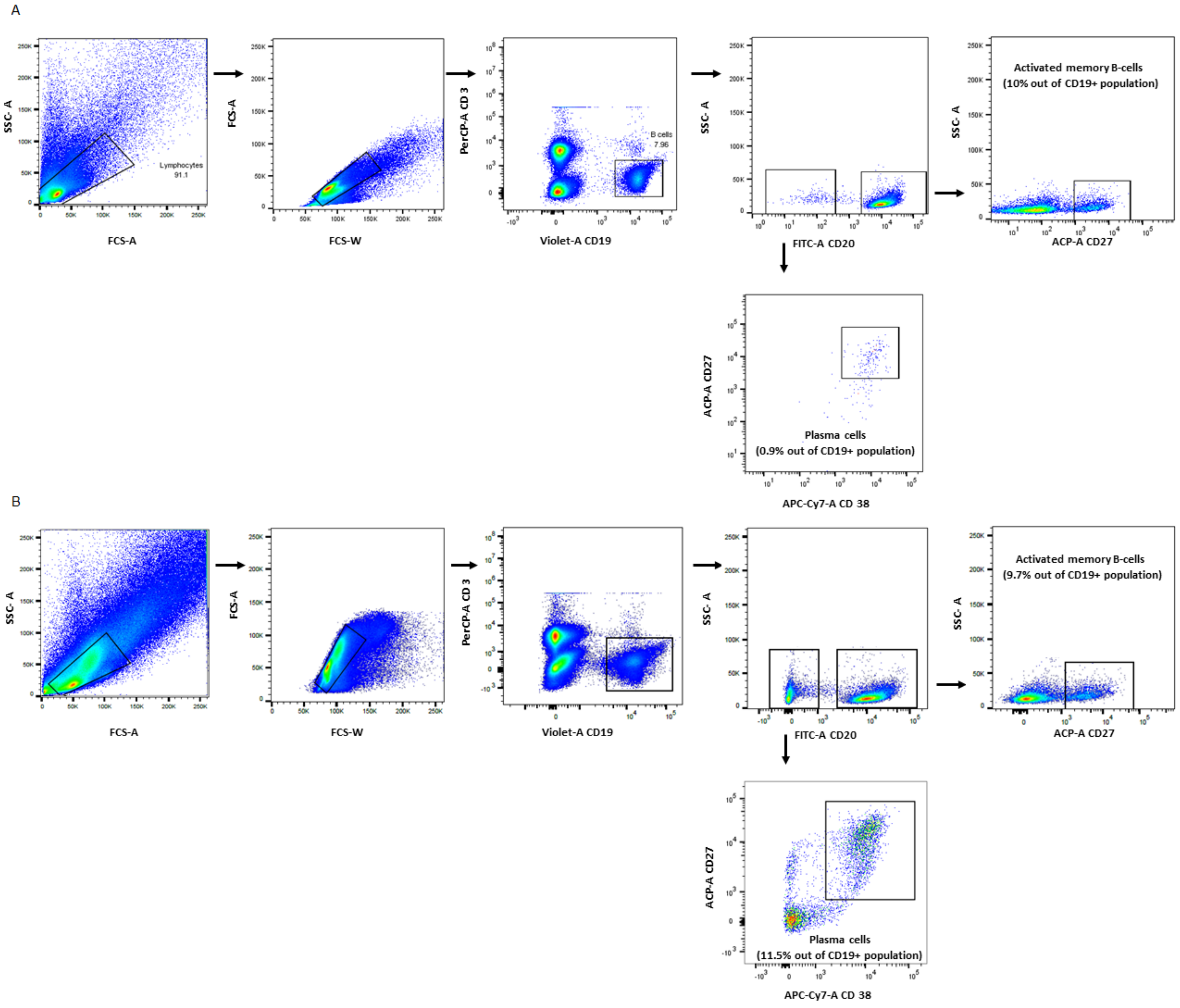

Figure S3: FACS of PB and mBC isolated from peripheral blood of IBD patient treated with IFX. (A) Gating on plasmablasts ($CD3^{-}CD19^{+}CD20^{-}CD27^{high}CD38^{high}$) and mBC ($CD3^{-}CD19^{+}CD20^{+}CD27^{+}$) mBC at D0. Approximately 0.9% of all B cells stained as PB, and 10% as mBC. (B) FACS of $CD3^{-}CD19^{+}CD20^{-}CD27^{high}CD38^{high}$ plasmablasts and $CD3^{-}CD19^{+}CD20^{+}$ mBC at D10. Approximately 11.5% of all B cells stained as PB, and 9.7% as mBC.

Table S1: List of all primers used for amplification of V_H genes. fw: forward, rev: reverse, UAd: universal adapter, Idx: index, RC: reverse complement.

PCR1

**IgH extansion forward**	**Extension + VH 5' specific region**
VH1-fw	CCCTCCTTTAATTCCC CAGGTCCAGCTKGTRCAGTCTGG
VH157-fw	CCCTCCTTTAATTCCC CAGGTGCAGCTGGTGSARTCTGG
VH3N-fw	CCCTCCTTTAATTCCC TCAACACAACGGTTCCCAGTTA
VH2-fw	CCCTCCTTTAATTCCC CAGRTCACCTTGAAGGAGTCTG
VH3-fw	CCCTCCTTTAATTCCC GAGGTGCAGCTGKTGGAGWCY
VH6-fw	CCCTCCTTTAATTCCC CAGGTACAGCTGCAGCAGTCA
VH4-fw	CCCTCCTTTAATTCCC CAGGTGCAGCTGCAGGAGTCS
VH4-DP63-fw	CCCTCCTTTAATTCCC CAGGTGCAGCTACAGCAGTGGG

**IgH extansion reverse**	**Extension (RC) + Ig constant specific region (RC)**
IgM-human-rev	GAGGAGAGAGAGAGAG GGTTGGGGCGGATGCACTCC
IgG-human-rev	GAGGAGAGAGAGAGAG SGATGGGCCCTTGGTGGARGC
IgA-human-rev	GAGGAGAGAGAGAGAG GGCTCCTGGGGGAAGAAGCC

PCR2

**IgALL universal forward**	**TruSeq universal adapter + Diversity region + Extension**
IgALL-UAd-fw	AATGATACGGCGACCACCGAGATCTACACTCTTTCCCTACACGACGCTCTTCCGATCT NNNN CCCTCCTTTAATTCCC

**IgALL index reverse**	**TruSeq universal adapter (RC) + Diversity region + Extension (RC)**
PE-Idx1-rev	CAAGCAGAAGACGGCATACGAGATCGTGATGTGACTGGAGTTCAGACGTGTGCTCTTCCGATCT NNNN GAGGAGAGAGAGAGAG
PE-Idx2-rev	CAAGCAGAAGACGGCATACGAGATACATCGGTGACTGGAGTTCAGACGTGTGCTCTTCCGATCT NNNN GAGGAGAGAGAGAGAG
PE-Idx3-rev	CAAGCAGAAGACGGCATACGAGATGCCTAAGTGACTGGAGTTCAGACGTGTGCTCTTCCGATCT NNNN GAGGAGAGAGAGAGAG
PE-Idx4-rev	CAAGCAGAAGACGGCATACGAGATTGGTCAGTGACTGGAGTTCAGACGTGTGCTCTTCCGATCT NNNN GAGGAGAGAGAGAGAG
PE-Idx5-rev	CAAGCAGAAGACGGCATACGAGATCACTGTGTGACTGGAGTTCAGACGTGTGCTCTTCCGATCT NNNN GAGGAGAGAGAGAGAG
PE-Idx6-rev	CAAGCAGAAGACGGCATACGAGATATTGGCGTGACTGGAGTTCAGACGTGTGCTCTTCCGATCT NNNN GAGGAGAGAGAGAGAG
PE-Idx7-rev	CAAGCAGAAGACGGCATACGAGATGATCTGGTGACTGGAGTTCAGACGTGTGCTCTTCCGATCT NNNN GAGGAGAGAGAGAGAG
PE-Idx8-rev	CAAGCAGAAGACGGCATACGAGATTCAAGTGTGACTGGAGTTCAGACGTGTGCTCTTCCGATCT NNNN GAGGAGAGAGAGAGAG
PE-Idx9-rev	CAAGCAGAAGACGGCATACGAGATCTGATCGTGACTGGAGTTCAGACGTGTGCTCTTCCGATCT NNNN GAGGAGAGAGAGAGAG
PE-Idx10-rev	CAAGCAGAAGACGGCATACGAGATAAGCTAGTGACTGGAGTTCAGACGTGTGCTCTTCCGATCT NNNN GAGGAGAGAGAGAGAG
